# Comparison and Evaluation of Different Radiotherapy Techniques Using Biodosimetry Based on Cytogenetics

**DOI:** 10.3390/cancers14010146

**Published:** 2021-12-29

**Authors:** Aggeliki Nikolakopoulou, Vasiliki Peppa, Antigoni Alexiou, George Pissakas, Georgia Terzoudi, Pantelis Karaiskos

**Affiliations:** 1 Laboratory of Health Physics, Radiobiology & Cytogenetics, Institute of Nuclear & Radiological Sciences & Technology, Energy & Safety, National Centre for Scientific Research ‘‘Demokritos’’, 15310 Athens, Greece; agg_nik@ipta.demokritos.gr (A.N.); gterzoudi@rrp.demokritos.gr (G.T.); 2 Medical Physics Laboratory, Medical School, National and Kapodistrian University of Athens, 11527 Athens, Greece; vpeppa@med.uoa.gr; 3 Radiotherapy Department, General Hospital of Athens Alexandra, 11528 Athens, Greece; antalexiou@hotmail.com (A.A.); pissakasg@gmail.com (G.P.)

**Keywords:** radiotherapy, radiobiological techniques, VMAT, biodosimetry, radiation dosimetry

## Abstract

**Simple Summary:**

Cell killing and tumor response in cancer patients depends not only on the absorbed radiation dose but also on the dose rate and delivery time. In this study, a biodosimetry assay based on the frequency of dicentrics chromosomes scored in peripheral blood lymphocytes from prostate cancer patients and PC3 human prostate cancer cell line was used to investigate the radiobiological impact of the relative prolonged dose delivery time and/or decreased dose rate met in advanced modulated radiotherapy techniques (VMAT and IMRT) compared to conventional non-modulated (3D-CRT) in prostate patient plan irradiations. The results showed a small but statistically significant decrease in the number of dicentrics following radiation with the modulated techniques, suggesting a corresponding decrease on the radiation dose efficiency. The biodosimetry assay could be used as an alternative to the laborious conventional clonogenic assay, while both lymphocytes and cancer cell line could effectively be used for estimation of the biological absorbed dose.

**Abstract:**

While rapid technological advances in radiotherapy techniques have led to a more precise delivery of radiation dose and to a decreased risk of side effects, there is still a need to evaluate the efficacy of the new techniques estimating the biological dose and to investigate the radiobiological impact of the protracted radiotherapy treatment duration. The aim of this study is to compare, at a cytogenetic level, advanced radiotherapy techniques VMAT and IMRT with the conventional 3D-CRT, using biological dosimetry. A dicentric biodosimetry assay based on the frequency of dicentrics chromosomes scored in peripheral blood lymphocytes from prostate cancer patients and PC3 human prostate cancer cell line was used. For each patient blood sample and each subpopulation of the cultured cell line, three different irradiations were performed using the 3D-CRT, IMRT, and VMAT technique. The absorbed dose was estimated with the biodosimetry method based on the induced dicentric chromosomes. The results showed a statistically significant underestimation of the biological absorbed dose of ~6% for the IMRT and VMAT compared to 3D-CRT irradiations for peripheral blood lymphocytes, whereas IMRT and VMAT results were comparable without a statistically significant difference, although slightly lower values were observed for VMAT compared to IMRT irradiation. Similar results were obtained using the PC3 cell line. The observed biological dose underestimation could be associated with the relative decreased dose rate and increase irradiation time met in modulated techniques compared to the conventional 3D-CRT irradiations.

## 1. Introduction

New technologies in radiotherapy techniques and treatment planning over the past few years had a great impact in the clinical outcome of cancer patients [1,2]. Radiation therapy (RT) is often used in conjunction with other therapies such as chemotherapy or resection as an integral part of both curative and palliative cancer treatments [1,3]. Modern radiotherapy techniques such as intensity-modulated radiation therapy (IMRT) and volumetric-modulated arc therapy (VMAT) have improved local tumor control with accuracy in dose delivery of the radiation, decreasing the dose of radiation to critical tissues near the tumor [1,2,3,4]. Moreover, both techniques have the potential to provide a safer dose escalation for a target [5,6] by increasing the radiation delivery time. Intensity-modulated radiation therapy (IMRT) uses radiation fields which are highly spatially and/or temporally regulated by modifying the beam shape and/or beam-on time, respectively. Volumetric-modulated arc therapy (VMAT) requires incessant gantry rotation and dynamic modulation of the dose rate [7]. Both the rate of monitor units per minute (MU rate) and collimation are varied over the course of a fraction, leading to different dose rates in every voxel of the calculation volume at any time during radiotherapy [8]. 

The dose rate is a factor of high importance in radiotherapy and radiobiology. It is well known that cell killing and tumor response in cancer patients depends not only on the absorbed radiation dose but also on the dose rate and delivery time. However, the biological response to dose rates of radiotherapy techniques is a controversial issue of the ongoing research [8,9]. The radiobiological impact of prolonged dose delivery time of a single fraction of advanced radiotherapy techniques, compared to conventional techniques with a shorter delivery time, has been extensively discussed in the literature [9,10,11,12]. Moreover, several studies of dose-rate effects on biological response showed that a prolonged delivery time of a single fraction considered less effective in terms of cell killing than shortened delivery time fractions [10,11,12,13,14]. Morgan et al. [14] showed that the longer dose delivery time involved in the IMRT technique is less biologically effective at killing cells than the conventional three-dimensional conformal radiotherapy (3D-CRT) technique. This effect may be the result of radiation-induced DNA damage repair during the extended radiation therapy. Furthermore, Bewes et al. [10] found that a longer delivery time can considerably increase the cell survival. Particularly, they showed that there is a statistically significant rise in clonogenic survival rates since the average dose rate was decreased for a standard total dose affecting radiation dose efficacy [10]. Moiseenko et al. [15] also showed that increasing the dose delivery time led to a statistically significant increase in cell survival for SiHa cells, using a clongenic assay, and supported the theory that DNA repair is related to the increase in survival rates detected when the dose delivery is increased [15]. Another in vitro study with hamster and human cell line demonstrated increased cell survivals for a 5 min IMRT head-and-neck treatment compared to a 75s acute radiation dose delivery showing that shortened fraction times can enhance radiation efficiency [16].

Both the IMRT and VMAT techniques use radiation fields which are highly spatially and/or temporally regulated by modifying beam shape and/or beam-on time, respectively. As a result, a wide range of dose rates is delivered to build up the planned dose distribution [8]. The differences in treatment technique or the number of beams used can radically affect the dose rate compared to conventional static non-modulated techniques due to the speed of modulation of the linear accelerator’s components [16]. Son et al. [17] assessed the impact of dose rate on normal tissues and the variation in the treatment efficacy for given energies and MU rates in VMAT technique. They showed that the dose volume parameter was independent of energy and MU rate and demonstrated the importance of evaluating the changes in dose rate in every voxel of the calculation volume during dose delivery. Podesta et al. [7] mapped the dose rates met in head and neck, lung, and prostate VMAT treatments and showed that up to 75% of the planning target volume (PTV) can have its dose delivered with dose rates of less than 1 Gy/min, with prostate plans on average demonstrating a lower mean dose rate within the PTV. Mackeprang et al. [8] observed clinical cases and illustrated that dose rates range over a continuous spectrum, with mean dose rates hardly exceeding 1Gy/min for conventional fractionation. 

The analysis of chromosome aberrations (CA) is considered one of the first implements to investigate the mechanisms of radiation action in human cells. The dicentric chromosome assay is the “gold standard” used in biological dosimetry in order to estimate biological absorbed dose by human lymphocytes [18]. Dicentric chromosomes are considered chromosome aberrations induced by ionizing radiation as a result of misrepair by nonhomologous end-joining whereby an exchange of material occurred on two damaged chromosomes [19]. In this study, a dicentric biodosimetry assay, based on the interpolation of the frequency of dicentrics chromosomes scored in peripheral blood lymphocytes, as well as to PC3 human prostate cancer cell line, to a pre-established dose effect calibration curve [20], was used to study the radiobiological impact of the relative prolonged dose-delivery time and/or decreased dose-rate met in advanced radiotherapy techniques (VMAT and IMRT) compared to 3D conformal radiation therapy (3D-CRT) in prostate patient plans. 

## 2. Materials and Methods

### 2.1. Cell Cultures and Ethics Statement for Human Data 

The peripheral blood samples (6 mL) obtained from nine prostate cancer patients before the first fraction of their treatment were drawn in heparinized tubes, and informed consent was obtained for each donor. The study was approved by the institutional Ethics Committee of the National and Kapodistrian University of Athens. Blood samples were transferred from the heparinized tubes to the Eppendorf safe-lock tubes 1.5 mL (colorless) and were located in a dedicated recess into a solid plate phantom. The same procedure was followed for subpopulations derived from the PC3 human prostate cancer cell line that was karyotypically tested. PC3 cell line was used in this study since it represents a good preclinical model of prostate tumor tissues.

### 2.2. Phantom Design and Blood Tube Housing

**Figure 1:**
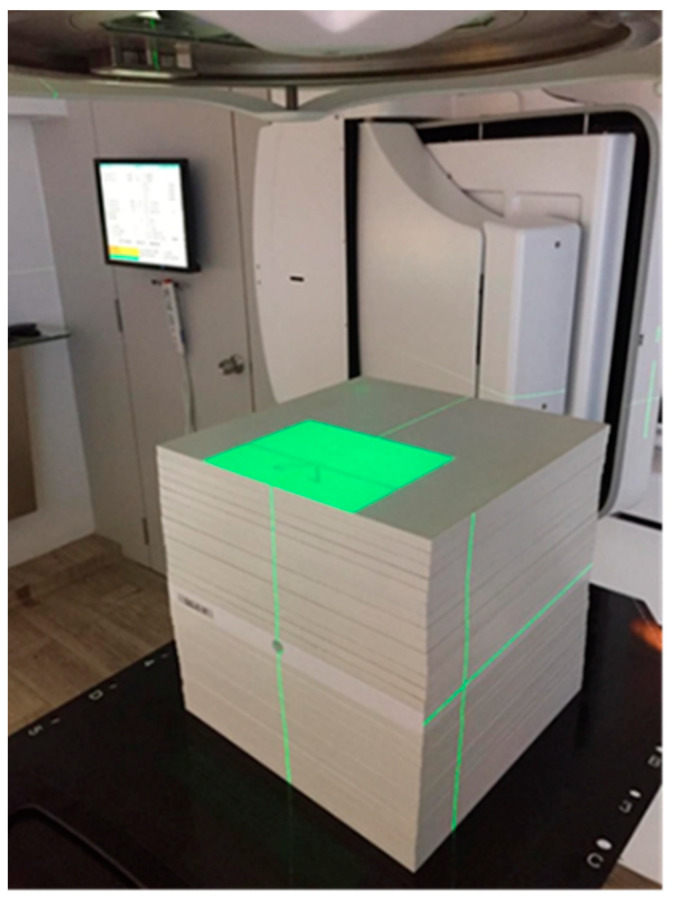
Picture of the 30 × 30 × 30 cm^3^ RW3 slab phantom incorporating the blood tube.

Figure 1 presents the white polystyrene (type RW3) solid plate phantom (IBA Dosimetry, Schwarzenbruck, Germany) used in this work. This consists of 28 plates of 1 cm thickness and the adapter plate of FC65-G detector (IBA Dosimetry, Schwarzenbruck, Germany) of 2 cm thickness, which was placed in the middle of the slabs, forming a 30 × 30 × 30 cm^3^ cube. The adapter plate was included in the phantom since it incorporates a cylindrical air-filled insert of 1.3 cm diameter suitable for the insertion of the blood tube (4 cm length/1.2 cm diameter), ensuring position stability and reproducibility during the irradiation.

### 2.3. Treatment Planning

A computational cube of 30 cm edge was included in the database of Monaco^®^ 5.11.02 (Elekta AB, Stockholm, Sweden) Treatment Planning System (TPS) in the form of CT image series in Digital Imaging and Communications in Medicine (DICOM) RT format. This model mimics the RW3 solid-plate phantom and was utilized for the generation of the treatment plans. A nominal relative electron density (RED) of 1.038 was assigned to the model to match the nominal RW3 mass density of 1.045 g/cc, according to the formulas used by Monaco^®^ TPS to convert RED to mass density. 

**Figure 2:**
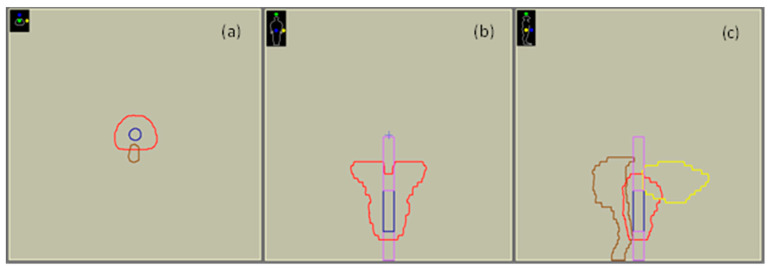
Screenshots from Monaco^®^ 5.11.02 TPS used for treatment planning and dose calculations presenting the computational model at the central axial (**a**) coronal (**b**) and sagittal (**c**) slice of the blood tube. Legend: red contour: PTV, brown contour: rectum, yellow contour: bladder, dark blue contour: blood tube, magenta contour: air-filled inserts.

Irradiation plans simulating a real prostate treatment were prepared. A structure set mimicking a real prostate case was generated (Figure 2) using the Monaco^®^ TPS-embedded tools. This set includes the planning target volume (PTV) consisting of the prostate gland and seminal vesicles, the rectum, and the bladder. The delineation procedure resulted in structure volumes of 172.2, 57.6, and 138.7 cc for the PTV, rectum, and bladder, respectively. A cylindrical region of interest (ROI) of 4 cm length and 1.2 cm diameter mimicking the blood tubes was also contoured within the PTV volume. A RED value of 1 was assigned to the blood tube, similar to that of water. Two cylindrical ROIs of 1.3 cm diameter and 4 and 7 cm length, respectively, were also delineated in either side of the tube to simulate the air-filled insert of the adapter plate with the blood tube incorporated (Figure 2). The RED value of air, equal to 0.001, was assigned to the air-filled cylinders.

Three treatment plans were performed using Monaco^®^ 5.11.02 treatment planning system (TPS): a 3D-CRT, an IMRT and a VMAT plan. All plans were created for delivery on the Infinity™ Linac (Elekta AB, Stockholm, Sweden) equipped with the Agility™ Multileaf Collimator (MLC) (Elekta AB, Stockholm, Sweden) of 160 leaves (spatial resolution of 5 mm at isocentre), using the 6 MV photon beam with a maximum dose rate of 600 MU/min. The prescribed dose was 76 Gy delivered in 2 Gy daily fractions. For all plans, the isocenter coincided with the geometric center of the tube. In order to minimize the potential dose deviations within the tube due to setup uncertainties, enhanced dose homogeneity within the PTV encompassing the blood tube as well as adequate PTV coverage had to be achieved through the treatment planning process. The tissue homogeneity was expressed by the Heterogeneity Index (HI), which is equal to (D5%)/(D95%), where D5% is the minimum dose delivered to the hottest 5% of the tissue and D95% is the minimum dose received by 95% of the tissue. (i.e., homogeneous dose distribution would generate an HI of 1). Treatment planning objectives regarding target coverage aimed a minimum dose of 95% of the prescribed dose to 95% of the PTV (D95% ≥ 95%) and a maximum dose of 105% of the prescribed dose to 2% of the PTV (D2% ≤ 105%). For all plans, organs at risk (OARs) constraints followed the recommendations of QUANTEC [21] for prostate radiotherapy. 

The 3D-CRT plan involved a four-field arrangement consisting of coplanar opposed anterior/posterior fields (0° and 180°) and left lateral/right lateral fields (90° and 270°) with a nominal dose rate of 600 MU/min. The inverse-planned IMRT plan included fixed gantry and intensity-modulated beams delivering the dose by means of the dynamic MLC approach. The plan was optimized using seven (0°, 55°, 90°, 125°, 235°, 270°, 305°) coplanar fields with a maximum number of 20 segments per field and with a nominal dose rate of 600 MU/min. The VMAT plan consisted of two coplanar arcs of 360° optimized simultaneously, to be delivered with opposite rotation (clock- and counter-clockwise), allowing the optimizer to achieve higher target homogeneity. The optimization process of the VMAT plan resulted in a total number of 163 segments. It should be mentioned that, for both IMRT and VMAT techniques, effort was made to push the optimization to the maximum achievable beyond reaching the planning objectives. For all plans, the dose to medium in medium (Dm, m) was calculated over the whole computational model geometry with a 3 mm grid resolution. The dose calculation for the 3D-CRT plan was performed using the collapsed cone algorithm, whereas the XVMC Monte Carlo dose engine [22,23] with a statistical uncertainty lower than 1% within the PTV was employed for the IMRT and VMAT plans. Dose calculations resulted in a total number of monitor units (MUs) equal to 314.58, 623.21, and 788.76 for the 3D- CRT, IMRT, and VMAT plan, respectively.

### 2.4. Irradiation Conditions

For each patient blood sample as well PC3 cells, three irradiations were performed in the Infinity™ Linac using the 3D-CRT, IMRT, and VMAT technique. In detail, the 30 × 30 × 30 cm^3^ RW3 phantom was placed on the treatment table of the Linac with the center of the tube coincided with the isocenter. The total beam-on time with respect to 3D-CRT, IMRT, and VMAT technique was 55, 121, and 165 s, respectively.

### 2.5. Dose Verification

In order to verify that the planned dose associated with the 3D-CRT, IMRT, and VMAT plan is accurately delivered to the cube, ionization chamber measurements were performed using a CC13 ionization chamber (IBA Dosimetry, Schwarzenbruck, Germany) placed at the same position within the 30 × 30 × 30 cm^3^ RW3 phantom with the tube. Three ion charge measurements were performed for each irradiation technique using the Dose1 electrometer (IBA Dosimetry, Schwarzenbruck, Germany). The mean charge value of each technique was converted to dose to water following the IAEA TRS-398 protocol [24] and measurements were compared with corresponding TPS calculations using the XVMC Monte Carlo dose engine with a dose grid resolution of 1 mm and a statistical uncertainty within the CC13 ROI lower than 1%. 

### 2.6. Blood Sample and Cell Line Culture—Biodosimetry Protocol 

After irradiation, all tubes with blood and cancer cells were delivered in temperature-controlled transport boxes (15–25 °C) within 30 min in the Laboratory of Health Physics, Radiobiology & Cytogenetics, INRASTES, “Demokritos”. The blood cultures were set by adding 1 mL of whole blood to 10 mL of Gibco^TM^ RPMI-1640 medium (Thermo Fisher Scientific, Waltham, Massachusetts, USA) supplemented with 10% fetal bovine serum, 1% PHA, 1% glutamine and antibiotics (penicillin: 10,000 U/mL; streptomycin: 10,000 μg/mL (Sigma-Aldrich, St. Louis, MO, USA)). PHA was dissolved in water at a concentration of 0.24 mg/mL. In continuous, cultures were incubated at 37 °C in a humidified incubator in an atmosphere of 5% CO_2_ and 95% air for 48 h. At approximately 45 h post irradiation, colcemid at a final concentration of 0.1µg/mL was added to cells 3 h prior fixation to increase and improve the quality and quantity of metaphases. Cells were collected by centrifugation, treated in 75 mM KCl for 10 min, fixed in methanol: glacial acetic acid 3:1 (*v*/*v*) and processed for cytogenetic analysis. Cells were spread on microscope slides, air dried, and stained in 3% Giemsa. Chromosomal damage was visualized and quantified as dicentrics in metaphase cells. For the chromosome analysis, the metaphases were located manually and their analysis was greatly facilitated by the use of a semi-automated image analysis system (IKAROS, MetaSystems, Germany [25]). For each experimental point, approximately 500 cells were scored for dicentric chromosome analysis, based on standard criteria. The specific method is stated in the IAEA technical report, 2001 [26].

PC3 cells were grown in 5 mL of D-MEM medium with 10% fetal bovine serum (FBS) and antibiotics at 37 °C in 5% CO_2_ and 95% air. PC3 attached cells were synchronized at their G0-phase and subsequently irradiated using the three radiotherapy techniques described earlier. Following exposure, cells were incubated at 37 °C for 24 h, and afterwards, colcemid at a final concentration of 0.1µg/mL was added to the cell cultures for five hours to accumulate metaphases for analysis. Cells were collected by trypsinization and centrifugation, treated in 75 mM KCl for 10 min and fixed in methanol:glacial acetic acid (3:1 *v*/*v*). Standard procedures [26] were used for chromosome preparation and giemsa staining, and chromosomal damage was visualized and quantified as dicentrics in metaphase cells. For each experimental point, approximately 500 cells were scored for chromosome damage based on standard criteria [27]. Using this experimental design, it was ensured that PC3 cells were proliferating and irradiated under exactly the same conditions.

In order to estimate the dose absorbed to both peripheral blood lymphocytes samples and PC3 cells the calibration curve of the laboratory was used, which is based on dicentric chromosomes and was generated by fitting the yield of aberrations to linear-quadratic dose dependencies. The radiation source used for establishing the calibration curve was Co-60 with a dose rate of 0.3 Gy/min. Based on the laboratory data the number of cells analysed was approximately 50,000. Biological absorbed dose estimations were performed with the free software CABAS V2.0 developed by Deperas et al. [28]. In particular, CABAS software consists of the main curve-fitting and dose-estimating module, as well as modules for calculating the dose in cases of partial body exposure.

### 2.7. Statistical Analysis

Differences in the biological absorbed dose for the peripheral blood lymphocytes as well as for PC3 cells between the 3D-CRT, IMRT, and VMAT irradiation techniques were evaluated for statistical significance using the Mann–Whitney U-test with a criterion of *p* ≤ 0.05. Mann–Whitney U Test was selected since the results of this work were not normally distributed according to the Komogorov–Smirnov test.

## 3. Results

### 3.1. Treatment Planning

**Figure 3:**
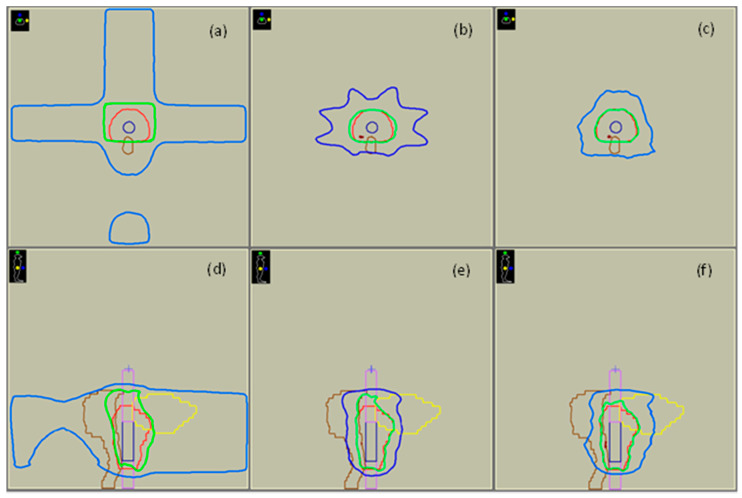
Screenshots from Monaco^®^ 5.11.02 TPS used for treatment planning and dose calculations presenting the isodose curves 105%, 95% and 50% of the prescribed dose superimposed on the central axial (**a**–**c**) and sagittal (**d**–**f**) slice of the blood tube for the 3D-CRT (left), IMRT (centre) and VMAT (right) treatment plan. Legend: red contour: PTV, brown contour: rectum, yellow contour: bladder, dark blue contour: blood tube, magenta contour: air-filled inserts, dark red line: 105% isodose, green line: 95% isodose, blue line: 50% isodose.

Figure 3 presents selected isodose lines of the 3D-CRT, IMRT and VMAT plan superimposed on the central axial and sagittal slice of the blood tube. Quantitative evaluation of plans by means of DVH metrics regarding PTV coverage (D95% and D2%), dose homogeneity (HI) and mean dose (D_mean_) for a single fraction is summarized in Table 1. In general, all plans respected the treatment planning objectives on target coverage and healthy tissues. Moreover, the mean dose and HI of the blood tube volumes were found to be within the 1% statistical uncertainty of corresponding MC TPS dose calculations, thus ensuring similar dose absorption independently of the radiotherapy technique used for the irradiation.

### 3.2. Dose Verification

Table 2 presents results of the comparison between TPS dose calculations for a single fraction delivery and corresponding CC13 ionization chamber measurements for the 3D-CRT, IMRT and VMAT plan verification. TPS calculations correspond to the mean dose of the delineated CC13 active volume obtained from the DVH statistics. An excellent agreement within 1% can be seen between TPS and experimental results for the 3D-CRT and IMRT technique. The maximum difference of 1.19% was observed for VMAT technique, however this discrepancy was marginally greater than the Monte Carlo statistical uncertainty of 1%; thus, it was not deemed important. 

### 3.3. Estimation of Biological Doses Based on Chromosome Aberration Analysis

**Figure 4:**
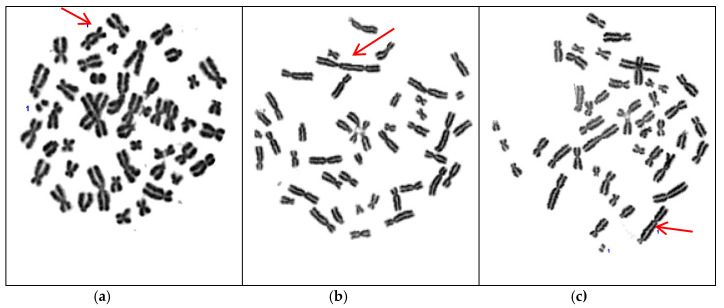
Representative examples of dicentric analysis at metaphase of peripheral blood lymphocytes, from a prostate cancer patient, irradiated using the (**a**) 3D-CRT, (**b**) IMRT, and (**c**) VMAT technique. The red arrows show the dicentric chromosomes.

In this study, the biological estimation of the absorbed dose, is based on the cytogenetic analysis of chromosomal aberrations such as radiation-induced dicentric chromosomes. Figure 4a–c show the metaphases with dicentric chromosomes in peripheral blood lymphocytes from a representative prostate cancer patient irradiated with the 3D-CRT, IMRT, and VMAT technique, respectively. 

**Figure 5:**
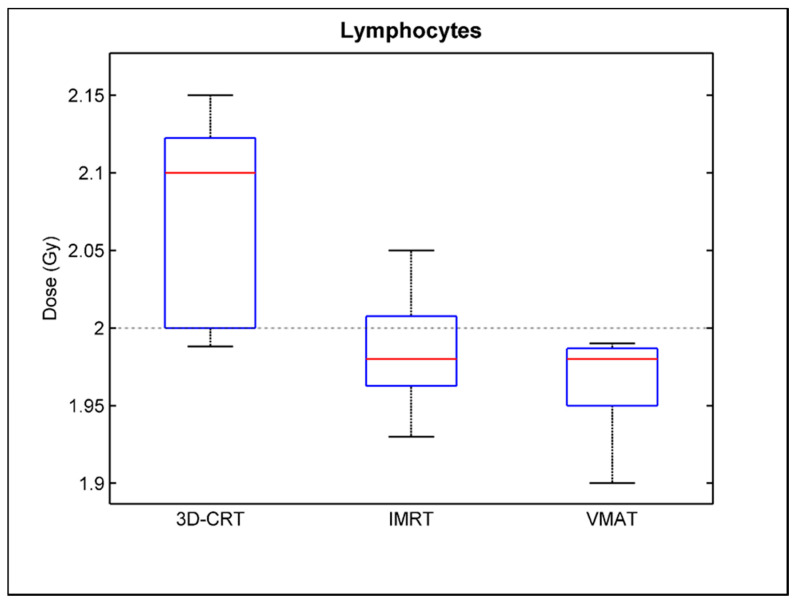
Box and whisker plots of the biological absorbed dose to the peripheral blood lymphocytes irradiated with the 3D-CRT, IMRT and VMAT technique across all prostate cancer patients.

**Figure 6:**
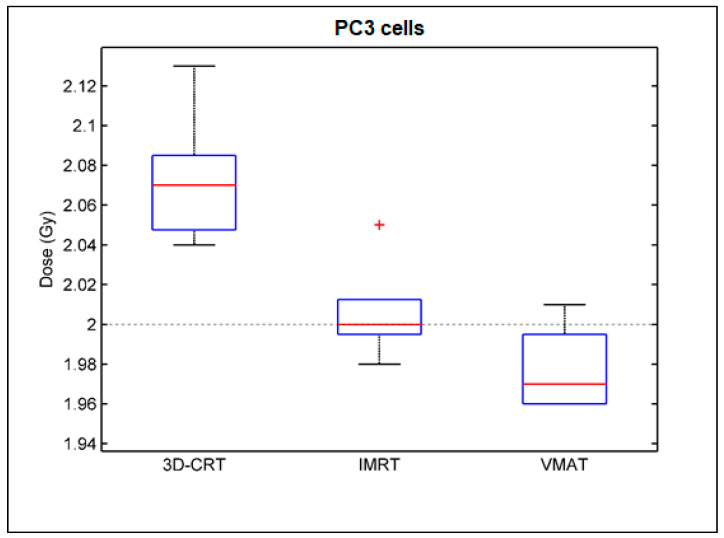
Box and whisker plots of the biological absorbed dose to the PC3 cells irradiated with the 3D-CRT, IMRT and VMAT technique.

Table 3 summarizes the comparison of the median biological absorbed dose between the 3D-CRT, IMRT, and VMAT technique for the peripheral blood lymphocytes of all prostate cancer patients as well as for the PC3 cells. It can be seen that the median biological absorbed dose associated with the blood lymphocytes was very similar to that of cancer cell line for each irradiation technique with differences not exceeding 1.4%. A statistically significant underestimation of the biological absorbed dose of ~6% for the IMRT and VMAT compared to 3D-CRT irradiations was observed for peripheral blood lymphocytes, whereas IMRT and VMAT results were comparable without a statistically significant difference (*p* > 0.05), although IMRT presents a relatively higher biological absorbed dose compared to VMAT for the majority of patient blood samples. Similar results presenting a statistically significant underestimation of the biological absorbed dose for the modulated techniques (IMRT and VMAT) were observed for the PC3 human prostate cancer cell line. This underestimation also can be observed in Figure 5 and Figure 6, which present the box plots of the biological absorbed dose derived for all lymphocytes and PC3 cells, respectively, for the 3D-CRT, IMRT, and VMAT irradiation technique, to demonstrate the variability of the biological dose values. It should be noted that the observed enhanced biological dose related to the 3D-CRT technique could not be attributed to differences in the dose delivered to the blood tube by each irradiation technique, since differences in the mean blood tube dose calculated by the TPS (Dmean) and verified by ionization chamber measurements between the 3D-CRT, IMRT, and VMAT technique were lower than 0.35% (Table 1).

## 4. Discussion

During an IMRT and especially during VMAT treatment, the dose to the target is delivered with a wide range of dose rates with a relatively high target volume, receiving the absorbed dose with dose rates less than 1 Gy/min [6,8,10,11,12], while the irradiation time is increased compared to conventional 3D-CRT irradiation. On the other hand, a concern of decreased bio-effectiveness has been reported when the tumor cells are irradiated with dose rates below 1 Gy/min [29].

Several studies have reported that recovery processes take place during irradiation when decreasing the dose rate and/or increasing the irradiation time that may reduce the therapeutic effect of radiation [30,31,32]. However, Oktaria et al. [4] reported inverse results demonstrated that the dose-rate effect appears to be inversed in their study. They demonstrated that mechanisms related to the inverse tenor of the dose-rate effect are not well documented but may be due to several reasons, such as the difference in radiosensitivity levels along the cell cycle at the time of irradiation [4]. Thus, cell survival data produced by uniform beam deliveries over a range of dose rates and exposure times may not always be precise in predicting the response to more complex radiotherapy techniques, such as IMRT and VMAT [33]. Therefore, it would be more relevant to investigate the biological effectiveness of the IMRT and VMAT techniques compared to conventional RT, by estimating the biological dose using biological dosimetry for irradiations similar to those performed for patient treatments with the different treatment techniques. 

In this study, the radiobiological impact of the relatively lower dose rate and higher dose-delivery time of a fractional dose of 2Gy for clinically used prostate patient plans using IMRT or VMAT technique compared to the conventional 3D-CRT was investigated by estimating the biological absorbed dose to the center of the target volume using the chromosomal aberrations of both human lymphocytes and cancer PC3 cell line evaluated by a dicentric biodosimetry assay. In the past few decades, cytogenetic biodosimetry has been applied for the estimation of the absorbed dose following exposure to ionizing radiation. In particular, the measurement of dicentrics has been used for more than three decades and is mentioned as the ‘gold’ standard method for biodosimetry [18]. In this study, frequencies of unstable chromosome aberrations (dicentrics, ring, and acentric fragments) were scored in metaphases of peripheral blood lymphocytes from prostate cancer patients and PC3 cells. Other chromosomal abnormalities such as centric and acentric rings were also observed and scored to support the extent of the damage. The data include the estimation of dose absorbed to peripheral blood lymphocytes and PC3 cell line using 3D-CRT, IMRT, and VMAT. The results showed a decrease in the number of dicentrics following radiation with the modulated IMRT and VMAT techniques compared to 3D-CRT irradiation, for the majority of prostate cancer patient blood samples leading to a statistically significant decrease of ~6% in the median biological dose absorbed for the same fractional dose of 2Gy. Similar results were obtained for the PC3 cells. These results could be associated with the lower dose-rate and the increased irradiation time met in IMRT and VMAT irradiations, since it is known that following DNA damage induced by irradiation, specific sensor proteins initiate a DNA damage response system including DNA damage repair within seconds [31]. Although previous studies [4,10] have evaluated, in vitro, the dose-rate effect on the biological effectiveness using the conventional clonogenic assay, biodosimetry assays could be used as an alternative to the laborious conventional clonogenic assay. The biodosimetry assay used in this study can be performed within 48 or 24 h for blood samples or cell lines, respectively, whereas more than two weeks are needed for the clonogenic assay. For future research, additional cell lines and tumor types could be tested, also cell mixtures and microenvironment models, using additional cytotoxicity analysis approaches for validation.

## 5. Conclusions

A dicentric biodosimetry assay based on the frequency of dicentrics chromosomes scored in peripheral blood lymphocytes from prostate cancer patients and PC3 human prostate cancer cell lines was used to derive biological absorbed doses and investigate the radiobiological impact of the relative prolonged dose-delivery time and/or decreased dose-rate met in advanced modulated radiotherapy techniques (VMAT and IMRT) compared to conventional 3D-CRT, in prostate radiotherapy treatments using typical patient plans. 

A statistically significant underestimation of the order of ~6% in the biological absorbed dose to both the peripheral blood lymphocytes and the PC3 cell lines for the modulated IMRT and VMAT compared to 3D-CRT irradiations was observed. Differences between the modulated techniques were not found to be statistically significant, although slightly lower values were observed for VMAT compared to IMRT irradiation. 

The observed biological dose underestimation could be associated with the relative decreased dose rate and increased irradiation time met in modulated techniques (IMRT and VMAT) compared to the conventional 3D-CRT irradiations.

## Figures and Tables

**Table 1:** DVH parameters for the PTV and tube volume obtained for the 3D-CRT, IMRT and VMAT plans.

Dosimetric Characteristic	Treatment Plan		
	3D-CRT	IMRT	VMAT
PTV			
D95% (%)	95.3	97.15	97.10
D2% (%)	102.5	104.6	104.3
D_mean_ (Gy)	1.988	2.013	2.008
HI	1.07	1.07	1.07
Blood tube			
D_mean_ (Gy)	1.993	2.000	1.998
HI	1.05	1.03	1.04

**Table 2:** Comparison of TPS calculations and corresponding CC13 measurements for the 3D-CRT, IMRT and VMAT plan.

Irradiation Technique	Dose (Gy)		% (D_TPS_/D_CC13_ − 1)
	TPS	CC13	
3D-CRT	2.013	1.995	0.90
IMRT	2.000	1.994	0.30
VMAT	1.993	2.017	−1.19

**Table 3:** Comparison of the median biological absorbed dose between the 3D-CRT, IMRT, and VMAT technique for the lymphocytes and PC3 cells.

Irradiation Technique	Median Value [min, max] (Gy)		Comparison	*p*-Value	
	Lymphocytes	PC3 Cells		Lymphocytes	PC3 Cells
3D-CRT	2.100 [1.988, 2.150]	2.070 [2.040, 2.130]	3D-CRT/IMRT	<0.05	<0.05
IMRT	1.980 [1.930, 2.050]	2.000 [1.980, 2.050]	IMRT/VMAT	0.3055	0.1270
VMAT	1.980 [1.900, 1.990]	1.970 [1.960, 2.010]	3D-CRT/VMAT	<0.05	<0.05

## Data Availability

The data presented in this study are available in this article.
